# Indocyanine green fluorescence angiography-guided simultaneous laparoscopic distal gastrectomy and spleen-preserving distal pancreatectomy for conserving the gastrosplenic ligament: A case report

**DOI:** 10.1016/j.ijscr.2022.106803

**Published:** 2022-01-29

**Authors:** Shun Kawaguchi, Satoshi Okubo, Shusuke Haruta, Junichi Shindoh, Masaji Hashimoto, Masaki Ueno

**Affiliations:** Department of Gastroenterological Surgery, Toranomon Hospital, Tokyo, Japan

**Keywords:** Indocyanine green, Gastrectomy, Pancreatectomy, ICG, indocyanine green, LDG, laparoscopic distal gastrectomy, LSPDP, laparoscopic spleen-preserving distal pancreatectomy, IPMN, intraductal papillary mucinous neoplasm, CT, computed tomography, MRCP, magnetic resonance cholangiopancreatography, DP, distal pancreatectomy, ShGA, short gastric arteries, POD, postoperative day, LIPA, left inferior phrenic artery, SpA, splenic artery, SpV, splenic vein, LGA, left gastric artery

## Abstract

**Introduction and importance:**

Indocyanine green (ICG) fluorescence angiography is being increasingly performed intraoperatively to detect restricted blood flow intraoperatively for the prevention of postoperative organ ischemia and anastomotic leakage. This is the first case report of simultaneous laparoscopic distal gastrectomy (LDG) and spleen-preserving distal pancreatectomy (LSPDP) involving ICG angiography use to avoid the remnant stomach ischemia.

**Case presentation:**

A 55-year-old man was diagnosed with early cancer of the stomach body and intraductal papillary mucinous neoplasms of the pancreatic tail. We performed simultaneous LDG with D2 dissection and LSPDP to conserve the gastrosplenic ligament and preserve blood supply to the remnant stomach. Intraoperatively, blood flow to the remnant stomach was visualized using ICG fluorescence angiography, after which Roux-en-Y reconstruction was performed. There was no perioperative remnant stomach ischemia.

**Clinical discussion:**

Despite the preserved splenic artery and vein, complete splenic infarction occurs after LSPDP possibly due to thrombus formation during surgical procedures. In this patient, we conserved the gastrosplenic ligament for the short gastric artery, which supplied blood to the remnant stomach; however, remnant stomach ischemia may occur. Therefore, we performed ICG fluorescence angiography during this operation to ensure that sufficient blood supply to the remnant stomach was maintained.

**Conclusion:**

Our experience demonstrates that ICG angiography may be useful for the prevention of remnant stomach ischemia.

## Highlights

• Remnant stomach ischemia could occur in simultaneous gastrectomy and pancreatectomy.
• Our patient had early gastric cancer and intraductal papillary mucinous neoplasm.
• He had laparoscopic distal gastrectomy and spleen-preserving distal pancreatectomy.
• Remnant stomach perfusion was visualized using indocyanine green angiography.
• Postoperative remnant stomach ischemia did not occur.

## 1 Introduction

Indocyanine green (ICG) is widely used to evaluate liver function. Intraoperative ICG angiography is being increasingly used to visualize the blood flow to target organs. Studies have suggested that by enabling clear visualization of the ischemic area, ICG reduces the rate of anastomotic leakage in patients undergoing colorectal surgery [1]. Similarly, ICG has also been used in gastric surgery to evaluate the blood flow to the remnant stomach [2].

Here, we report a case of successful simultaneous laparoscopic distal gastrectomy (LDG) and laparoscopic spleen-preserving distal pancreatectomy (LSPDP) to conserve the splenic artery and vein and gastrosplenic ligament and prevent stomach ischemia through visualization of its blood supply using ICG fluorescence angiography.

This case report is reported in line with the SCARE 2020 criteria [3].

## 2 Case presentation

**Fig. 1:**
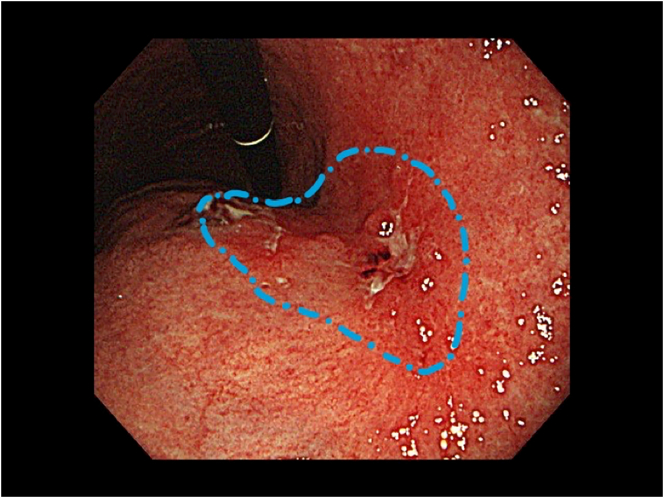
Gastric endoscopy image shows findings indicative of early gastric cancer at the lesser curvature of the stomach (blue circle). (For interpretation of the references to colour in this figure legend, the reader is referred to the web version of this article.)

**Fig. 2:**
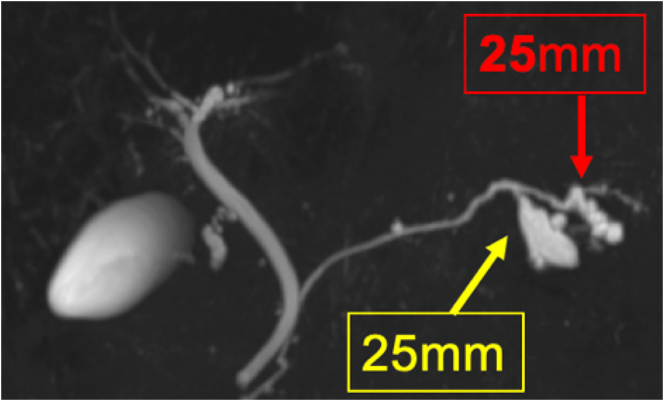
Magnetic resonance cholangiopancreatography image. The arrows indicate two intraductal papillary mucinous neoplasms.

Early gastric cancer was incidentally detected in a 55-year-old man during a regular medical checkup. His medical history included type 2 diabetes mellitus and intraductal papillary mucinous neoplasm (IPMN). Histopathological examination of the endoscopic biopsy specimen revealed a 55-mm, moderately to poorly differentiated adenocarcinoma at the lesser curvature of the stomach (Fig. 1). Computed tomography (CT) and magnetic resonance cholangiopancreatography (MRCP) revealed no evidence of metastasis (N0 and M0); however, two multilocular cystic lesions connected to the main pancreatic duct at the pancreatic tail were observed, without main pancreatic duct dilation but the solid component within the cyst was enhanced and the cyst wall was thickened. Subsequent MRCP revealed a gradual increase in the diameter of one of the lesions (from 18 mm to 25 mm over 3 years; Fig. 2). The patient was diagnosed with a branch-duct type IPMN with a low malignant potential according to the 2017 international guidelines for IPMN. The levels of serum tumor markers, such as carcinoembryonic antigen and carbohydrate antigen 19-9, were within the normal range.

**Fig. 3:**
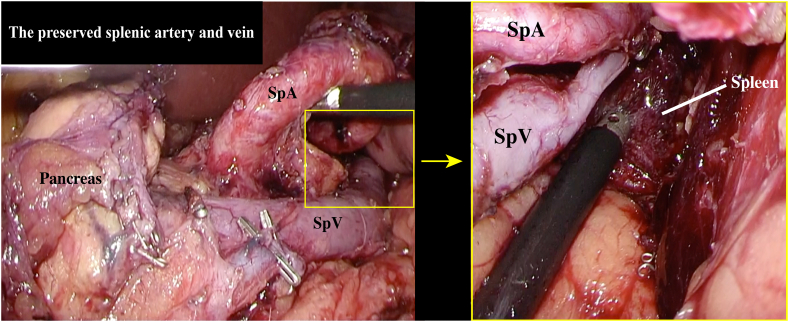
Image showing the preserved splenic artery and vein after laparoscopic distal gastrectomy with D2 dissection and distal pancreatectomy. The body and tail of the pancreas and the distal stomach have been removed. SpA: splenic artery, SpV: splenic vein, LGA root: clipped root of the left gastric artery.

**Fig. 4:**
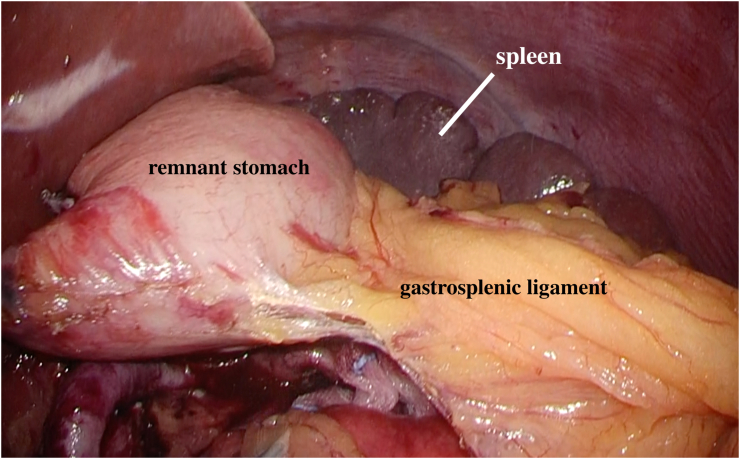
Image displaying the preserved gastrosplenic ligament after laparoscopic distal gastrectomy with D2 dissection and distal pancreatectomy but before Roux-en-Y reconstruction.

**Fig. 5:**
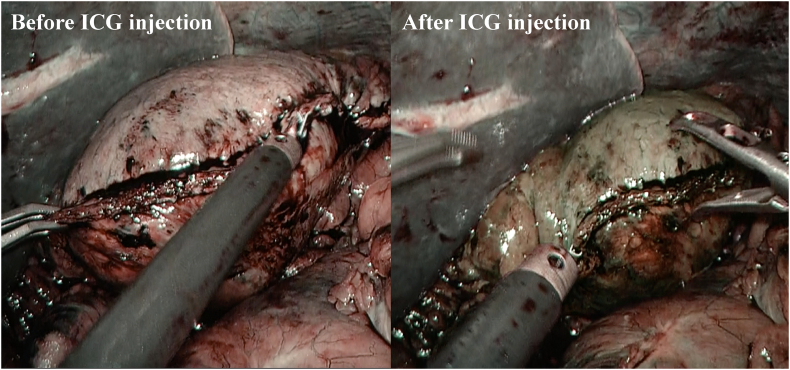
Indocyanine green fluorescence angiography image showing the blood flow to the remnant stomach after distal gastrectomy and distal pancreatectomy but before Roux-en-Y reconstruction is seen. (For interpretation of the references to colour in this figure legend, the reader is referred to the web version of this article.)

Although there were no features suggestive of IPMN malignancy, considering the patient's young age and the difficulty in performing distal pancreatectomy (DP) after gastrectomy, simultaneous LDG and LSPDP with Roux-en-Y reconstruction were performed. To avoid gastric necrosis, we planned to preserve the gastrosplenic ligament and splenic vessels, including the short gastric arteries (ShGA), during LSPDP, and performed ICG angiography to visualize the blood flow to the remnant stomach. First, we performed LDG with D2 lymph node dissection and completed the LSPDP procedure (Fig. 3), while preserving the gastrosplenic ligament (Fig. 4). Next, we injected 7.5 mg of ICG (Dianogreen; Dai-Ichi Pharm, Tokyo, Japan) and assessed blood flow to the remnant stomach using a 1588 AIM camera system with endoscopic near-infrared visualization (Stryker Corporation, Kalamazoo, USA) (Fig. 5). We then performed Roux-en-Y reconstruction. The total operation time was 555 min, and the blood loss was 512-ml.

Postoperatively, the patient developed a pancreatic fistula, which was conservatively treated with abdominal drainage tube insertion. On postoperative day (POD) 29, the tube was removed, and the patient was discharged on POD 32. Histopathological examination revealed poorly differentiated gastric adenocarcinoma, well-differentiated gastric adenocarcinoma, and intraductal papillary mucinous adenoma. The overall stage of these cancers was T1b, N0, M0 (stage IA) according to the 8th edition of the American Joint Committee on Cancer gastric cancer TNM staging system. CT and fiber gastroscopy revealed no evidence of recurrence or splenic artery or vein thrombosis at 46 months postoperatively.

## 3 Discussion

Ischemic necrosis of the remnant stomach after gastrectomy was first reported in the 1950s [4]. The stomach has a good compensatory blood supply; however, gastric ischemia may occur due to vascular endothelial injury or thrombus formation during surgery. Gastric ischemia can also occur during gastrectomy performed after other abdominal surgeries, and can have lethal outcomes. To prevent ischemia, an understanding of the gastric vasculature and intraoperative blood flow to the stomach is helpful [5]. Blood supply to the remnant stomach of our patient, who simultaneously underwent LDG and LSPDP for conserving the gastrosplenic ligament, mainly depended on two arteries [5]: the ShGA via the splenic artery and the left inferior phrenic artery (LIPA). However, Hasegawa et al. reported that LIPA alone may not provide supply sufficient blood to the remnant stomach [6]. In contrast, a previous case report described successful distal gastrectomy (DG) after traditional DP combined with splenectomy in which there was no postoperative remnant stomach necrosis despite blood supply to the gastric remnant through the LIPA alone [7]. This was because ShGA had been resected 11 years before DG was performed, and collateral flow through the LIPA had been augmented. Our patient did not have good collateral blood flow to the remnant stomach from the LIPA. Therefore, we decided to preserve the gastrosplenic ligament and splenic vessels, including the ShGA, during LSPDP.

Since the first report of laparoscopic DP in 1996, modified surgical techniques that preserve the spleen and splenic vessels have been developed. LSPDP is being increasingly performed [8], [9], especially for treating low-grade malignancies and benign tumors of the pancreatic tail. The LSPDP procedure in which the splenic artery and vein are conserved is known as Kimura's method, and reports suggest that it has the advantage of a lower occurrence rate of pancreatic fistula and splenic infarction than traditional DP with splenectomy [10]. The use of Kimura's method made it possible to perform LSPDP with LDG without remnant stomach ischemia [11], [12], [13]. However, complete splenic infarction has been reported to have occurred in a patient who underwent LSPDP with Kimura's method [14], possibly due to thrombus formation resulting from intraoperative compression of the arterial wall. In this patient, a microthrombus from the splenic artery may have influenced blood flow through the downstream vessels, such as ShGA. Therefore, assessing the blood flow to the remnant stomach using ICG fluorescence angiography is important for this patient.

ICG is gaining popularity as a tool for its cost-effectiveness and minimally invasive intraoperative angiography [15]. It is considered more objective and reliable than empirical intraoperative evaluation by surgeons [16]. Various quantitative methods of intraoperatively assessing bowel viability have been investigated; however, most of them lack good-quality evidence supporting their use [17], [18]. Meanwhile, ICG angiography has been proven to be effective for the prevention of anastomotic leakage and postoperative organ ischemia not only in colorectal surgeries [1], [19], but also in gastric surgeries [2], [20]. ICG angiography can be effective in confirming the remnant stomach perfusion during simultaneous LDG and LSPDP, as highlighted in our case.

## 4 Conclusion

We report the first case of simultaneous LDG and LSPDP in which the gastrosplenic ligament was conserved and ICG angiography of the remnant stomach was performed. The use of ICG can be considered a safe strategy for assessing blood flow to the remnant stomach, although further quantitative evaluation is required.

## Sources of funding

None.

## Ethical approval

Ethics committee approval was not required given the article type (case report).

## Consent

Written informed consent was obtained from the patient for publication of this case report and any accompanying images. A copy of the written consent is available for review by the Editor-in-Chief of this Journal on request.

## Authorship declaration

All authors revised the manuscript, approved the manuscript to be published, and agreed to be accountable for all aspects of the work and for ensuring that questions related to the accuracy or integrity of any part of the work are appropriately investigated and resolved.

## Author contribution

Shun Kawaguchi: data collection, data analysis, drafting, writing the paper, revising, final approval.

Satoshi Okubo: operator, concept, revising.

Shusuke Haruta: operator, revising.

Junichi Shindoh: revising.

Masaji Hashimoto: revising.

Masaki Ueno: revising.

## Research registration number

The research registry number of this case report was 7524.

## Guarantor

The Guarantor for this study is Shun Kawaguchi MD.

## Provenance and peer review

Not commissioned, externally peer-reviewed.

## Declaration of competing interest

None.
